# [*N*-((*E*)-2-{[2-(Dimethyl­amino)­eth­yl]imino­meth­yl}phen­yl)-*N*-(2,6-dimethyl­phen­yl)anilinido-κ^3^ *N*,*N*′,*N*′′]ethyl­zinc

**DOI:** 10.1107/S1600536811005514

**Published:** 2011-02-23

**Authors:** Mathurin Issa-Madongo, Ying Mu

**Affiliations:** a State Key Laboratory of Supramolecular Structure and Materials, Jilin University, Changchun 130012, People’s Republic of China

## Abstract

The title eth­yl–zinc complex, [Zn(C_2_H_5_)(C_19_H_24_N_3_)], bears a tridentate anilinide–aldimine ligand and features one long Zn—N(amine) bond length, attributable to the crowded environment of the coordinated metal, arising from the dimethyl­phenyl group. The Zn^II^ ion adopts a distorted tetra­hedral geometry, the dihedral angle between the two benzene rings being 86.05 (16)°.

## Related literature

For the synthesis, luminescent properties and applications in catalysis of complexes bearing anilido–aldimine ligands, see: Liu *et al.* (2005 ▶, 2006 ▶); Ren *et al.* (2007 ▶); Su *et al.* (2007 ▶); Yao *et al.* (2008 ▶).
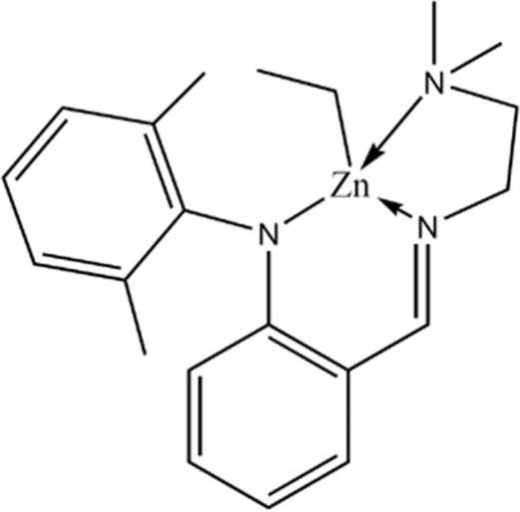


## Experimental

### 

#### Crystal data

[Zn(C_2_H_5_)(C_19_H_24_N_3_)]
*M* *_r_* = 388.84
Monoclinic, 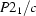

*a* = 7.3664 (15) Å
*b* = 13.849 (3) Å
*c* = 20.296 (4) Å
β = 99.26 (3)°
*V* = 2043.5 (7) Å^3^
*Z* = 4
Mo *K*α radiation
μ = 1.21 mm^−1^
*T* = 293 K
0.23 × 0.21 × 0.19 mm

#### Data collection

Rigaku R-AXIS RAPID diffractometer
Absorption correction: multi-scan (*ABSCOR*; Higashi, 1995 ▶) *T*_min_ = 0.768, *T*_max_ = 0.803
18874 measured reflections
4571 independent reflections
2619 reflections with *I* > 2σ(*I*)
*R*_int_ = 0.116

#### Refinement

*R*[*F* ^2^ > 2σ(*F* ^2^)] = 0.071
*wR*(*F* ^2^) = 0.189
*S* = 0.96
4571 reflections
231 parameters
H-atom parameters constrained
Δρ_max_ = 0.98 e Å^−3^
Δρ_min_ = −0.63 e Å^−3^

### 

Data collection: *RAPID-AUTO* (Rigaku, 1998 ▶); cell refinement: *RAPID-AUTO*; data reduction: *CrystalStructure* (Rigaku/MSC, 2002 ▶); program(s) used to solve structure: *SHELXS97* (Sheldrick, 2008 ▶); program(s) used to refine structure: *SHELXL97* (Sheldrick, 2008 ▶); molecular graphics: *SHELXTL* (Sheldrick, 2008 ▶); software used to prepare material for publication: *SHELXL97*.

## Supplementary Material

Crystal structure: contains datablocks global, I. DOI: 10.1107/S1600536811005514/bh2336sup1.cif

Structure factors: contains datablocks I. DOI: 10.1107/S1600536811005514/bh2336Isup2.hkl

Additional supplementary materials:  crystallographic information; 3D view; checkCIF report

## Figures and Tables

**Table 1:** Selected bond lengths (Å)

Zn1—C20	1.991 (5)
Zn1—N1	2.009 (3)
Zn1—N2	2.487 (4)
Zn1—N3	2.004 (4)

**Fig. 1.**
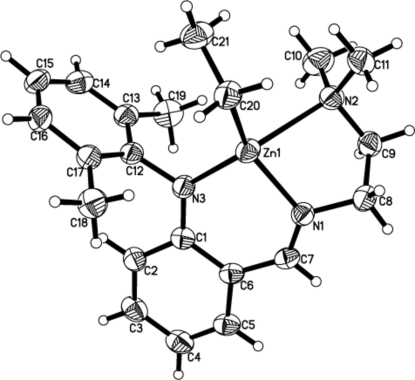
View of the title complex, with displacement ellipsoids drawn at the 30% probability level.

## supplementary crystallographic information

### Comment

Complexes bearing chelating anilido-aldimine ligands have recently attracted attention because such ligands have similar framework and combine the steric and electronic characteristics of β-diketiminate and salicyaldiminato ligands, which have been extensively researched in coordination chemistry and catalysis. We have recently reported the luminescent properties of a series of Al(III) (Liu *et al.*, 2005, 2006), Zn^II^ (Su *et al.*, 2007) and B(III) (Ren *et al.*, 2007) complexes with chelating anilido-aldimine ligands, and catalytic properties of Al(III) complexes (Yao *et al.*, 2008) for the polymerization of ε-caprolactone. Good results have been obtained. As a part of our study, the preparation and crystal structure of the title ethyl zinc complex bearing *N*,*N'*,*N''*-tridentate anilido-aldimine ligand is reported.

In the complex (Fig. 1), the C7═N1 [1.292 (6) Å] bond length is comparable to those found in similar anilido-imine compounds (Su *et al.*, 2007). The bond distances Zn—N1 [2.009 (3) Å] and Zn—N3 [2.004 (4) Å] are similar to those in other anilido-aldimine zinc complexes. The pendant arm bond length Zn—N2 [2.487 (4) Å], in contrast, is longer than equivalent bonds found in other *N*,*N'*,*O*-tridentate Schiff base Zn complexes. This may be due to the crowded environment in the metal center arising from the aryl group bonded to N3. The dihedral angle between the central benzene ring (C1···C6) and the benzene ring (C12···C17) is 86.05 (16)°.

### Experimental

The complex was synthesized according to the literature method (Su *et al.*, 2007). A solution of ZnEt_2_ (1.0 mol/*L*, 2.0 mmol) was added to a solution of the ligand (0.59 g, 2.0 mmol) in toluene (30 ml) at 273 K. After stirring for 24 h, the solution was evaporated to dryness and the resulting yellow powder was washed with hexane (Yield: 0.65 g, 90%). Anal. Calcd. for C_21_H_29_N_3_Zn (388.87): C 64.86, H 7.52, N 10.81%; Found: C 64.62, H 7.44, N 10.65%.

### Refinement

The C-bound H atoms were positioned geometrically with C—H = 0.93 (aromatic and imine carbon), 0.97 (methylene) and 0.96 Å (methyl), and allowed to ride on their parent atoms with *U*_iso_(H) = 1.2 (1.5 for methyl) *U*_eq_(C).

### Crystal data

**Table:**

[Zn(C_2_H_5_)(C_19_H_24_N_3_)]	*F*(000) = 824
*M**_r_* = 388.84	*D*_x_ = 1.264 Mg m^−^^3^
Monoclinic, *P*2_1_/*c*	Mo *K*α radiation, λ = 0.71073 Å
Hall symbol: -P 2ybc	Cell parameters from 10491 reflections
*a* = 7.3664 (15) Å	θ = 6.2–54.9°
*b* = 13.849 (3) Å	µ = 1.21 mm^−^^1^
*c* = 20.296 (4) Å	*T* = 293 K
β = 99.26 (3)°	Block, yellow
*V* = 2043.5 (7) Å^3^	0.23 × 0.21 × 0.19 mm
*Z* = 4	

### Data collection

**Table:**

Rigaku R-AXIS RAPID diffractometer	4571 independent reflections
Radiation source: fine-focus sealed tube	2619 reflections with *I* > 2σ(*I*)
graphite	*R*_int_ = 0.116
ω scans	θ_max_ = 27.5°, θ_min_ = 3.1°
Absorption correction: multi-scan (*ABSCOR*; Higashi, 1995)	*h* = −9→9
*T*_min_ = 0.768, *T*_max_ = 0.803	*k* = −16→17
18874 measured reflections	*l* = −24→26

### Refinement

**Table:**

Refinement on *F*^2^	Primary atom site location: structure-invariant direct methods
Least-squares matrix: full	Secondary atom site location: difference Fourier map
*R*[*F*^2^ > 2σ(*F*^2^)] = 0.071	Hydrogen site location: inferred from neighbouring sites
*wR*(*F*^2^) = 0.189	H-atom parameters constrained
*S* = 0.96	*w* = 1/[σ^2^(*F*_o_^2^) + (0.097*P*)^2^] where *P* = (*F*_o_^2^ + 2*F*_c_^2^)/3
4571 reflections	(Δ/σ)_max_ = 0.023
231 parameters	Δρ_max_ = 0.98 e Å^−^^3^
0 restraints	Δρ_min_ = −0.63 e Å^−^^3^
0 constraints	

### Fractional atomic coordinates and isotropic or equivalent isotropic displacement parameters (Å^2^)

**Table:**

	*x*	*y*	*z*	*U*_iso_*/*U*_eq_	
C1	0.7787 (7)	0.8763 (3)	0.8847 (2)	0.0497 (10)	
C2	0.7141 (7)	0.8858 (3)	0.8154 (2)	0.0558 (11)	
H2	0.7400	0.8373	0.7866	0.067*	
C3	0.6141 (8)	0.9649 (4)	0.7893 (3)	0.0672 (14)	
H3	0.5740	0.9683	0.7435	0.081*	
C4	0.5719 (8)	1.0393 (4)	0.8297 (3)	0.0671 (14)	
H4	0.5068	1.0931	0.8115	0.080*	
C5	0.6275 (7)	1.0321 (3)	0.8964 (3)	0.0580 (12)	
H5	0.5944	1.0808	0.9237	0.070*	
C6	0.7337 (6)	0.9537 (3)	0.9266 (2)	0.0483 (10)	
C7	0.7748 (7)	0.9542 (3)	0.9974 (2)	0.0512 (11)	
H7	0.7175	1.0017	1.0190	0.061*	
C8	0.8819 (8)	0.8962 (3)	1.1069 (2)	0.0581 (12)	
H8A	0.8122	0.9508	1.1194	0.070*	
H8B	1.0066	0.9005	1.1310	0.070*	
C9	0.7926 (8)	0.8012 (3)	1.1237 (2)	0.0594 (12)	
H9A	0.7967	0.7965	1.1716	0.071*	
H9B	0.6646	0.8003	1.1027	0.071*	
C10	0.7658 (10)	0.6345 (4)	1.0890 (3)	0.090 (2)	
H10A	0.8310	0.5805	1.0745	0.135*	
H10B	0.6627	0.6499	1.0554	0.135*	
H10C	0.7229	0.6183	1.1299	0.135*	
C11	1.0496 (9)	0.6923 (4)	1.1502 (3)	0.0737 (16)	
H11A	1.0089	0.6737	1.1910	0.111*	
H11B	1.1306	0.7468	1.1584	0.111*	
H11C	1.1138	0.6393	1.1339	0.111*	
C12	0.9206 (7)	0.7257 (3)	0.8645 (2)	0.0505 (10)	
C13	0.8071 (8)	0.6443 (3)	0.8554 (2)	0.0580 (12)	
C14	0.8532 (8)	0.5707 (3)	0.8151 (2)	0.0662 (14)	
H14	0.7762	0.5174	0.8068	0.079*	
C15	1.0110 (9)	0.5750 (3)	0.7871 (3)	0.0701 (16)	
H15	1.0435	0.5233	0.7621	0.084*	
C16	1.1190 (9)	0.6548 (4)	0.7958 (2)	0.0667 (14)	
H16	1.2234	0.6577	0.7755	0.080*	
C17	1.0774 (8)	0.7330 (3)	0.8347 (2)	0.0579 (12)	
C18	1.1964 (9)	0.8214 (4)	0.8426 (3)	0.0718 (15)	
H18A	1.1236	0.8770	0.8275	0.108*	
H18B	1.2934	0.8144	0.8165	0.108*	
H18C	1.2486	0.8294	0.8887	0.108*	
C19	0.6367 (9)	0.6368 (4)	0.8876 (3)	0.0818 (17)	
H19A	0.5704	0.5792	0.8724	0.123*	
H19B	0.5598	0.6921	0.8758	0.123*	
H19C	0.6714	0.6342	0.9353	0.123*	
C20	1.2613 (7)	0.7185 (3)	1.0170 (3)	0.0611 (12)	
H20A	1.3406	0.7502	0.9898	0.073*	
H20B	1.3175	0.7252	1.0634	0.073*	
C21	1.2474 (9)	0.6109 (3)	0.9989 (3)	0.0777 (16)	
H21A	1.1714	0.5788	1.0263	0.117*	
H21B	1.3680	0.5827	1.0062	0.117*	
H21C	1.1941	0.6038	0.9528	0.117*	
N1	0.8827 (6)	0.8962 (2)	1.03518 (17)	0.0499 (9)	
N2	0.8894 (6)	0.7183 (2)	1.10019 (19)	0.0564 (10)	
N3	0.8810 (6)	0.8002 (2)	0.91016 (18)	0.0520 (9)	
Zn1	1.01743 (8)	0.78379 (3)	1.00320 (2)	0.0531 (2)	

### Atomic displacement parameters (Å^2^)

**Table:**

	*U*^11^	*U*^22^	*U*^33^	*U*^12^	*U*^13^	*U*^23^
C1	0.040 (3)	0.051 (2)	0.058 (3)	0.0013 (17)	0.007 (2)	0.0038 (17)
C2	0.052 (3)	0.063 (2)	0.053 (3)	0.007 (2)	0.010 (2)	0.0040 (19)
C3	0.055 (4)	0.080 (3)	0.066 (3)	0.017 (3)	0.008 (3)	0.021 (2)
C4	0.052 (4)	0.068 (3)	0.083 (4)	0.021 (2)	0.018 (3)	0.023 (2)
C5	0.045 (3)	0.052 (2)	0.079 (3)	0.0110 (19)	0.015 (2)	0.009 (2)
C6	0.039 (3)	0.043 (2)	0.064 (3)	0.0028 (16)	0.011 (2)	0.0048 (17)
C7	0.046 (3)	0.043 (2)	0.066 (3)	0.0022 (17)	0.012 (2)	−0.0009 (18)
C8	0.060 (4)	0.059 (3)	0.055 (3)	0.003 (2)	0.008 (2)	−0.0090 (19)
C9	0.060 (4)	0.064 (3)	0.056 (3)	0.006 (2)	0.015 (2)	0.002 (2)
C10	0.100 (6)	0.065 (3)	0.106 (5)	−0.013 (3)	0.016 (4)	−0.008 (3)
C11	0.076 (5)	0.084 (3)	0.058 (3)	0.022 (3)	−0.001 (3)	0.006 (2)
C12	0.051 (3)	0.050 (2)	0.048 (2)	0.0102 (18)	0.002 (2)	0.0017 (17)
C13	0.057 (4)	0.057 (2)	0.056 (3)	0.010 (2)	−0.001 (2)	0.0015 (19)
C14	0.067 (4)	0.055 (3)	0.069 (3)	0.003 (2)	−0.011 (3)	−0.003 (2)
C15	0.093 (5)	0.055 (3)	0.059 (3)	0.020 (3)	0.002 (3)	−0.010 (2)
C16	0.074 (4)	0.072 (3)	0.056 (3)	0.025 (3)	0.015 (3)	0.002 (2)
C17	0.064 (4)	0.058 (3)	0.050 (3)	0.010 (2)	0.004 (2)	0.0031 (18)
C18	0.065 (4)	0.073 (3)	0.078 (4)	−0.002 (3)	0.016 (3)	0.000 (2)
C19	0.065 (5)	0.081 (3)	0.096 (4)	−0.004 (3)	0.003 (3)	−0.001 (3)
C20	0.051 (3)	0.069 (3)	0.061 (3)	0.014 (2)	0.002 (2)	−0.004 (2)
C21	0.073 (5)	0.066 (3)	0.089 (4)	0.018 (3)	−0.001 (3)	−0.003 (2)
N1	0.050 (3)	0.0491 (18)	0.050 (2)	0.0020 (15)	0.0082 (17)	−0.0017 (14)
N2	0.056 (3)	0.056 (2)	0.056 (2)	0.0045 (17)	0.0019 (19)	−0.0002 (16)
N3	0.055 (3)	0.0526 (18)	0.047 (2)	0.0103 (16)	0.0023 (17)	−0.0017 (14)
Zn1	0.0500 (4)	0.0525 (3)	0.0541 (3)	0.0111 (2)	0.0003 (2)	−0.0051 (2)

### Geometric parameters (Å, °)

**Table:**

C1—N3	1.349 (5)	C12—C17	1.391 (7)
C1—C2	1.417 (6)	C12—C13	1.398 (7)
C1—C6	1.441 (6)	C12—N3	1.447 (5)
C2—C3	1.376 (6)	C13—C14	1.383 (6)
C2—H2	0.9300	C13—C19	1.510 (8)
C3—C4	1.384 (8)	C14—C15	1.375 (8)
C3—H3	0.9300	C14—H14	0.9300
C4—C5	1.353 (7)	C15—C16	1.356 (8)
C4—H4	0.9300	C15—H15	0.9300
C5—C6	1.418 (6)	C16—C17	1.404 (6)
C5—H5	0.9300	C16—H16	0.9300
C6—C7	1.419 (6)	C17—C18	1.499 (7)
C7—N1	1.292 (6)	C18—H18A	0.9600
C7—H7	0.9300	C18—H18B	0.9600
C8—N1	1.457 (5)	C18—H18C	0.9600
C8—C9	1.533 (6)	C19—H19A	0.9600
C8—H8A	0.9700	C19—H19B	0.9600
C8—H8B	0.9700	C19—H19C	0.9600
C9—N2	1.471 (6)	C20—C21	1.534 (6)
C9—H9A	0.9700	Zn1—C20	1.991 (5)
C9—H9B	0.9700	C20—H20A	0.9700
C10—N2	1.470 (7)	C20—H20B	0.9700
C10—H10A	0.9600	C21—H21A	0.9600
C10—H10B	0.9600	C21—H21B	0.9600
C10—H10C	0.9600	C21—H21C	0.9600
C11—N2	1.472 (7)	Zn1—N1	2.009 (3)
C11—H11A	0.9600	Zn1—N2	2.487 (4)
C11—H11B	0.9600	Zn1—N3	2.004 (4)
C11—H11C	0.9600		
			
N3—C1—C2	122.1 (4)	C13—C14—H14	119.4
N3—C1—C6	121.5 (4)	C16—C15—C14	119.9 (4)
C2—C1—C6	116.4 (4)	C16—C15—H15	120.0
C3—C2—C1	122.0 (4)	C14—C15—H15	120.0
C3—C2—H2	119.0	C15—C16—C17	121.7 (5)
C1—C2—H2	119.0	C15—C16—H16	119.1
C2—C3—C4	121.5 (5)	C17—C16—H16	119.1
C2—C3—H3	119.3	C12—C17—C16	117.4 (5)
C4—C3—H3	119.3	C12—C17—C18	121.7 (4)
C5—C4—C3	118.5 (4)	C16—C17—C18	120.9 (5)
C5—C4—H4	120.8	C17—C18—H18A	109.5
C3—C4—H4	120.8	C17—C18—H18B	109.5
C4—C5—C6	123.1 (4)	H18A—C18—H18B	109.5
C4—C5—H5	118.5	C17—C18—H18C	109.5
C6—C5—H5	118.5	H18A—C18—H18C	109.5
C5—C6—C7	116.6 (4)	H18B—C18—H18C	109.5
C5—C6—C1	118.6 (4)	C13—C19—H19A	109.5
C7—C6—C1	124.7 (4)	C13—C19—H19B	109.5
N1—C7—C6	127.6 (4)	H19A—C19—H19B	109.5
N1—C7—H7	116.2	C13—C19—H19C	109.5
C6—C7—H7	116.2	H19A—C19—H19C	109.5
N1—C8—C9	107.1 (4)	H19B—C19—H19C	109.5
N1—C8—H8A	110.3	C21—C20—Zn1	112.5 (4)
C9—C8—H8A	110.3	C21—C20—H20A	109.1
N1—C8—H8B	110.3	Zn1—C20—H20A	109.1
C9—C8—H8B	110.3	C21—C20—H20B	109.1
H8A—C8—H8B	108.6	Zn1—C20—H20B	109.1
N2—C9—C8	110.5 (4)	H20A—C20—H20B	107.8
N2—C9—H9A	109.6	C20—C21—H21A	109.5
C8—C9—H9A	109.6	C20—C21—H21B	109.5
N2—C9—H9B	109.6	H21A—C21—H21B	109.5
C8—C9—H9B	109.6	C20—C21—H21C	109.5
H9A—C9—H9B	108.1	H21A—C21—H21C	109.5
N2—C10—H10A	109.5	H21B—C21—H21C	109.5
N2—C10—H10B	109.5	C7—N1—C8	119.6 (4)
H10A—C10—H10B	109.5	C7—N1—Zn1	125.3 (3)
N2—C10—H10C	109.5	C8—N1—Zn1	113.8 (3)
H10A—C10—H10C	109.5	C10—N2—C9	110.2 (4)
H10B—C10—H10C	109.5	C10—N2—C11	108.7 (4)
N2—C11—H11A	109.5	C9—N2—C11	110.1 (4)
N2—C11—H11B	109.5	C10—N2—Zn1	118.1 (3)
H11A—C11—H11B	109.5	C9—N2—Zn1	103.7 (2)
N2—C11—H11C	109.5	C11—N2—Zn1	105.7 (3)
H11A—C11—H11C	109.5	C1—N3—C12	118.0 (4)
H11B—C11—H11C	109.5	C1—N3—Zn1	127.9 (3)
C17—C12—C13	121.4 (4)	C12—N3—Zn1	113.6 (3)
C17—C12—N3	119.6 (4)	C20—Zn1—N3	119.23 (18)
C13—C12—N3	118.8 (4)	C20—Zn1—N1	142.01 (18)
C14—C13—C12	118.3 (5)	N3—Zn1—N1	91.01 (14)
C14—C13—C19	120.3 (5)	C20—Zn1—N2	99.88 (18)
C12—C13—C19	121.3 (4)	N3—Zn1—N2	126.21 (16)
C15—C14—C13	121.1 (5)	N1—Zn1—N2	75.94 (13)
C15—C14—H14	119.4		
